# Plasma neurofilament light chain in relation to 10-year change in cognition and neuroimaging markers: a population-based study

**DOI:** 10.1007/s11357-023-00876-5

**Published:** 2023-08-03

**Authors:** Joyce van Arendonk, Frank J. Wolters, Julia Neitzel, Elisabeth J. Vinke, Meike W. Vernooij, Mohsen Ghanbari, M. Arfan Ikram

**Affiliations:** 1 Department of Radiology and Nuclear Medicine, Erasmus MC-University Medical Center Rotterdam, Rotterdam, the Netherlands; 2 Department of Epidemiology, Erasmus MC-University Medical Center Rotterdam, PO Box 2040, Rotterdam, 3000 CA the Netherlands; 3 Department of Epidemiology, Harvard T.H Chan School of Public Health, Boston, MA USA

**Keywords:** Neurofilament light chain, Cognition, Neuroimaging, Population-based

## Abstract

Neurofilament light chain (NfL) is a promising biomarker for risk stratification and disease monitoring of dementia, but its utility in the preclinical disease stage remains uncertain. We determined the association of plasma NfL with (change in) neuroimaging markers and cognition in the population-based Rotterdam Study, using linear and logistic regression and mixed-effects models. Plasma NfL levels were measured using the Simoa NF-light™ assay in 4705 dementia-free participants (mean age 71.9 years, 57% women), who underwent cognitive assessment and brain MRI with repeated assessments over a 10-year follow-up period. Higher plasma NfL was associated with worse cognitive performance at baseline (g-factor: *β* =  − 0.12 (− 0.15; − 0.09), *p* < 0.001), and accelerated cognitive decline during follow-up on the Stroop color naming task (*β* = 0.04 (0.02; 0.06), *p* < 0.001), with a smaller trend for decline in global cognition (g-factor *β* =  − 0.02 (− 0.04; 0.00), *p* = 0.044). In the subset of 975 participants with brain MRI, higher NfL was associated with poorer baseline white matter integrity (e.g., global mean diffusivity: *β* = 0.12 (0.06; 0.19), *p* < 0.001), with similar trends for volume of white matter hyperintensities (*β* = 0.09 (0.02; 0.16), *p* = 0.011) and presence of lacunes (OR = 1.55 (1.13; 2.14), *p* = 0.007). Plasma NfL was not associated with volumes or thickness of the total gray matter, hippocampus, or Alzheimer signature regions. In conclusion, higher plasma NfL levels are associated with cognitive decline and larger burden of primarily white matter pathology in the general population.

**Supplementary Information:**

The online version contains supplementary material available at 10.1007/s11357-023-00876-5.

## Introduction

Dementia is a clinical syndrome that is often preceded by a long preclinical phase, during which various types of neuropathology may accumulate [1]. Timely and effective preventive interventions against dementia require easily obtainable biomarkers that reflect the underlying processes in the preclinical phase. Neurofilament light chain (NfL) offers promise as a sensitive and easily obtainable biomarker of neurodegeneration, due partly to high correlations between levels of NfL in plasma and cerebrospinal fluid [2]. NfL is an intermediary filament of the neuronal cytoskeleton that is released in the cerebrospinal fluid and blood upon axonal damage. We have previously shown that high levels of plasma NfL are associated with an increased risk of developing dementia, including clinical Alzheimer’s disease (AD), in the general population [3]. However, the extent to which NfL is associated with cognitive function and decline in the preclinical disease stage, in participants without a clinical diagnosis of dementia, remains uncertain. While some studies found a significant association between plasma NfL and cognitive performance or decline in participants free of dementia [4–8], others did not [9, 10] or observed these associations only in patients with mild cognitive impairment [11, 12]. These inconsistencies might be driven by differences in sample size (ranging from 38 to 602), study population (clinic-based versus population-based), and follow-up time (ranging from 2 to 7 years).

In addition to clinical outcome, an understanding of how NfL reflects different types of brain pathology can help to determine its suitability for monitoring different therapeutic target effects. Several brain imaging studies have attempted to characterize the pathology underlying abnormal plasma levels of NfL using brain MRI markers of neurodegeneration or cerebrovascular disease. On the basis of its axonal origin, one would expect the strongest associations of NfL with markers of white matter injury (e.g., white matter hyperintensities (WMH) and microstructural integrity) rather than gray matter pathology. Prior imaging studies, however, show contrasting results. While some population-based studies of healthy participants found associations of plasma NfL with gray matter volume cross-sectionally [13–15], another did not [6], and only in two out of four studies this was consistent with an association between NfL and gray matter atrophy over time [6, 14–16]. Similarly, regarding white matter pathology, population-based studies generally observed associations of NfL with the presence of WMH but not with change in WMH volume over time [6, 13–15]. Microstructural integrity using DTI may be more sensitive to detect change in subclinical white matter pathology over time [15, 16], but the association of NfL with the microstructure across various brain regions and tracts is undetermined.

We therefore determined the association of plasma NfL levels with change in cognitive function and brain imaging markers of gray and white matter pathology over a 10-year follow-up period in the population-based Rotterdam Study.

## Methods

### Study design and participants

**Fig. 1:**
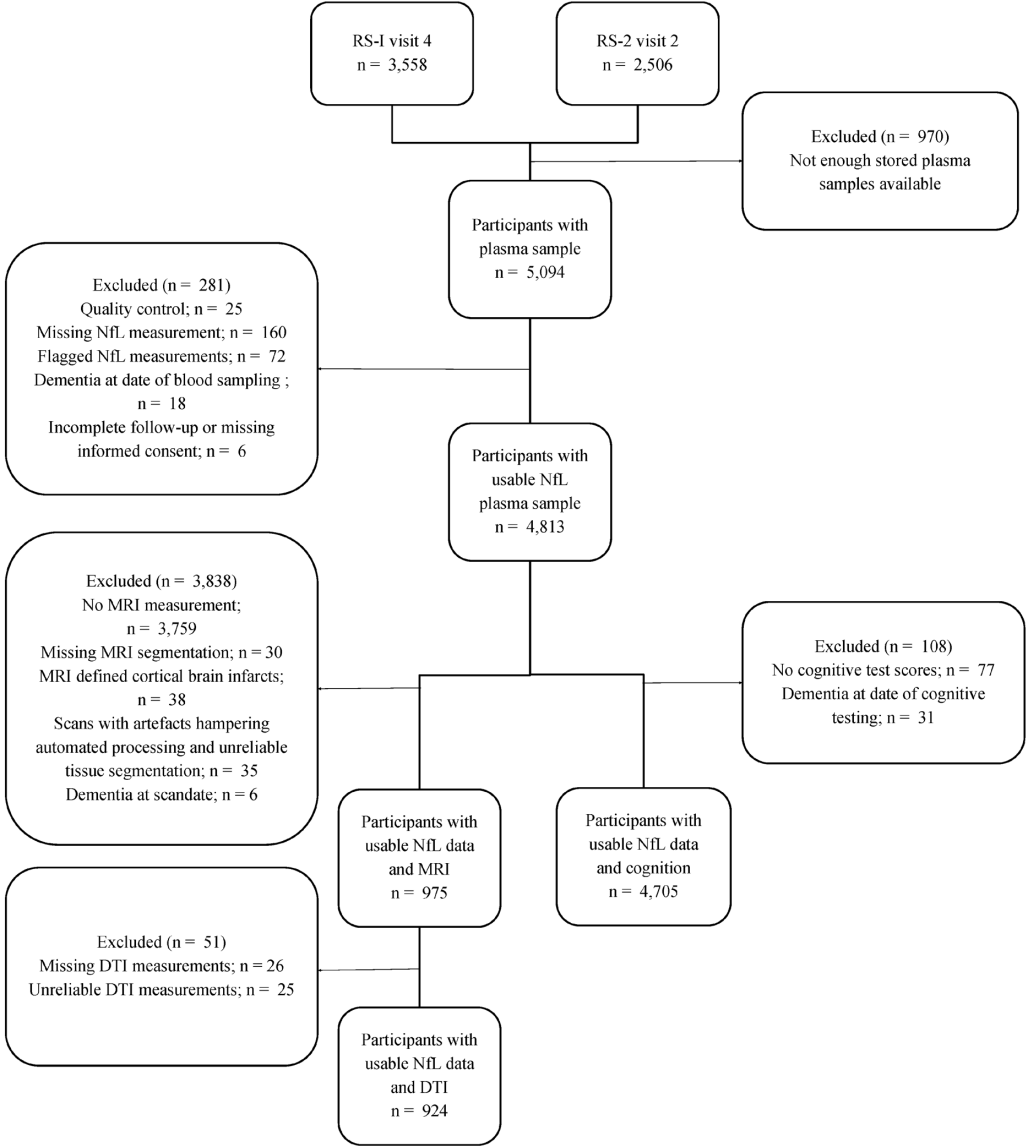
Flowchart of the inclusion of participants with usable plasma neurofilament light chain (NfL) values and MRI imaging data or cognitive test scores available. Abbreviations: *DTI* diffusion tension imaging, *MRI* magnetic resonance imaging, *RS-1* Rotterdam Study first cohort, *RS-2* Rotterdam Study second cohort

The present study is embedded in the Rotterdam Study, a population-based prospective cohort study that aims to assess the determinants and occurrence of age-related diseases [17]. The original cohort started in 1990 with 7983 inhabitants aged 55 and older (RS-I) from the Ommoord area, a suburb of Rotterdam. In 2000, the cohort was extended with a new wave of 3011 participants who had reached age 55 years or moved into the study area (RS-II). In-person examinations are repeated every 4 years. For the current study, we included all participants who attended the fourth visit of RS-I and the second visit of RS-II (Fig. 1, Supplementary Fig. 1). Between 2002 and 2005, 5094 participants had blood samples taken during their visit to the study center. We excluded 257 participants with missing or invalid test results for plasma NfL levels, 18 participants with dementia at the time of blood sampling, and 6 participants who withdrew informed consent for further follow-up. Dementia was assessed at each study visit and through continuous follow-up screening; details are described in the supplementary methods. Of the remaining 4813 participants, 4705 underwent detailed cognitive assessment at baseline, of which 3045 participants had at least one repeated cognitive measurement.

From 2005 onwards, all participants were invited for routine brain magnetic resonance imaging (MRI) as part of the core Rotterdam Study protocol [18]. Of all 4705 participants in this study between 2002 and 2005, a subset of 1048 participants without dementia underwent MRI (response rate: 89%). Scans with cortical infarcts (*n* = 38) or other causes of failed automated processing and unreliable tissue segmentation (*n* = 35) were excluded, leaving 975 participants in the analyses of an association between NfL and MRI volumetric measures, of which 808 participants had more than 1 scan. Moreover, 51 participants had either missing or low-quality DTI measurements at baseline, leaving 924 participants in the cross-sectional analyses of microstructural white matter integrity. For the longitudinal DTI analyses, 1022 participants were included with repeated DTI measurements using the later implemented new protocol.

### Ethics

The Rotterdam Study has been approved by the Medical Ethics Committee of the Erasmus MC (registration number MEC 02.1015) and by the Dutch Ministry of Health, Welfare and Sport (Population Screening Act WBO, license number 1071272–159,521-PG). The Rotterdam Study Personal Registration Data collection is filed with the Erasmus MC Data Protection Officer under registration number EMC1712001. The Rotterdam Study has been entered into the Netherlands National Trial Register (NTR; www.trialregister.nl) and into the WHO International Clinical Trials Registry Platform (ICTRP; https://apps.who.int/trialsearch/) under shared catalog number NL6645/NTR6831. All participants provided written informed consent to participate in the study and to have their information obtained from treating physicians.

### Assessment of plasma concentrations of NfL

According to standard procedures, ethylenediamine tetra-acetic-treated (EDTA) plasma was sampled, aliquoted, and frozen at − 80 °C. From plasma samples collected between 2002 and 2005, NfL measurements were assessed in two separate batches (5094 measurements) on a single molecule array (Simoa) HD-1 analyzer platform [19] with the NF-light advantage kit [20] at Quanterix (Lexington, MA, USA). The first batch of 2000 samples was obtained from a random selection of 1000 participants from the fourth visit of RS-I and 1000 from the second visit of RS-II. The second batch of 3094 samples was obtained from the remaining participants of these two study waves. Samples were tested in duplicate, and on each plate, two quality control samples were run. NfL was measured with the NF-light advantage kit [20]. The coefficient of variation for all available plasma samples is published elsewhere [3]. Participant’s data were excluded from the analyses when the concentration coefficient of variation exceeded 20%, when control samples were out of range, or when the duplicates or single measurements were missing.

### MRI acquisition and processing

Multi-sequence brain MRI was performed on a single 1.5 T MRI scanner (GE Signa Excite). Imaging included T1-weighted, proton density-weighted, fluid-attenuated inversion recovery (FLAIR), T2*-weighted sequences, and diffusion tensor imaging (DTI). The detailed imaging protocol can be found elsewhere [18] and in the supplementary methods. Imaging was done at baseline (mean difference from time of blood sampling: 1.4 (SD 1) years) and at three follow-up examinations (after a mean follow-up of 3.5 (SD 0.6), 5.9 (SD 1.3), 10.0 (SD 0.4) years). The scanner and imaging protocols were kept identical throughout the study duration, except for a change in the DTI sequence. To assure comparability of longitudinal measures, only DTI data acquired with the newest protocol were included in the longitudinal DTI analyses (see supplementary Fig. 1).

For tissue segmentation, images were segmented into cerebrospinal fluid (CSF), gray matter, normal-appearing white matter [21], and white matter hyperintensities [22] using an automated processing algorithm. In brief, k-nearest-neighbor classification was automated by non-rigidly registering MR data with a tissue probability atlas. More details are described in the supplementary methods. All segmentations were manually checked and corrected if necessary [18]. Total brain volume was calculated as the sum of gray matter, normal-appearing white matter, and white matter hyperintensities (WMH) volume. White matter was the sum of normal-appearing white matter and WMH volumes. Supratentorial intracranial volume, as a proxy for head size, was calculated by summing CSF volumes to the total brain volume. Hippocampal volume and cortical thickness were obtained by processing T1-weighted MR images with FreeSurfer (version 6.0). The volumes of the left and right hippocampi were subsequently summed. We used the mean cortical thickness and the surface area-weighted average of two previously defined composite signatures of AD cortical thinning based on work by Jack and colleagues [23] (ADsig Mayo) and by Dickerson and colleagues (ADsig Harvard) [24]. The ADsig Mayo comprises temporal regions, including the entorhinal cortex, fusiform, inferior, and middle temporal regions. The ADsig Harvard comprises frontal, temporal, and parietal regions, including the entorhinal cortex, parahippocampus, inferior parietal lobe, pars opercularis, pars orbitalis, pars triangularis, inferior temporal, temporal pole, precuneus, supramarginal gyrus, superior parietal, and superior frontal regions. Diffusion data included global mean fractional anisotropy (FA) and mean diffusivity (MD) in the normal-appearing white matter and were preprocessed using a standardized processing pipeline, which is described in more detail in the supplementary methods [25]. Using a probabilistic diffusion tractography approach, 15 different white matter tracts (12 tracts were present in both left and right hemispheres) were segmented [26]. Participant-specific mean FA and MD inside each white matter tract were obtained, and left and right measures were averaged. We combined the tissue and tract segmentations to obtain tract-specific white matter volumes and tract-specific WMH volumes (natural log-transformed to account for their skewed distribution). See supplementary methods for further details. Tracts were categorized based on anatomy or presumed function into brainstem tracts (middle cerebellar peduncle, medial lemniscus), projection tracts (corticospinal tract, anterior thalamic radiation, superior thalamic radiation, posterior thalamic radiation), association tracts (superior longitudinal fasciculus, inferior longitudinal fasciculus, inferior fronto-occipital fasciculus, uncinate fasciculus), limbic system tracts (cingulate gyrus part of cingulum, parahippocampal part of cingulum and fornix), and callosal tracts (forceps major, forceps minor) [26].

Trained research physicians, blinded to NfL measurements, visually rated all scans for the presence, number, and location of infarcts and cerebral microbleeds [18]. Cortical infarcts were rated as focal lesions with tissue loss of cortical gray matter and same signal intensity as CSF on all sequences and a hyperintense rim on FLAIR when located supratentorially. Subcortical lesions ≥ 3 mm and ≤ 15 mm in size were classified as lacunar infarcts. Focal round to ovoid areas < 10 mm of very low signal intensity on T2*-weighted imaging were rated as microbleeds.

### Cognitive assessment

Participants underwent an extensive cognitive assessment, comprising the Stroop test (error adjusted time in seconds taken for completing a reading, color naming, and interference task), the Purdue pegboard test (PPT, sum score of three trials), the 15-word learning test (15-WLT, total number of words remembered immediately and at 10 min after immediate recall), the word fluency test (WFT, amount of animals named within 60 s), and the letter-digit substitution task (LDST, number of correct digits within 60 s) [27]. We calculated a measure of global cognition (g-factor) by extracting the first component of a principal component analysis (PCA) based on the above-mentioned cognitive tests. We only included the interference subtask for the Stroop test and the delayed recall subtask for the 15-WLT, because high correlations between subtasks within a single test could lead to distortion of the factor loadings. Cognitive function was assessed at baseline (i.e., time of blood sampling) and at two subsequent follow-up examinations (after a mean follow-up of 6.5 (SD 0.4) and 11.1 (SD 0.5) years, respectively).

### Assessment of covariates

Blood pressure was measured twice in sitting position at the right arm, and the average of these two measurements was used. Smoking habits, level of education, and medication use, including blood pressure-lowering medication, lipid-lowering medication, and antidiabetics, were assessed by interview. Educational attainment was categorized into lower (primary, unfinished secondary, and lower vocational), further (secondary or intermediate vocational education), and higher education (higher vocational education or university). Smoking habits were categorized as current, former, and never smokers. Hypertension was defined as a blood pressure > 140/90 mmHg or the use of blood pressure-lowering medication [28]. Blood samples were drawn to assess levels of cholesterol, high-density lipoprotein (HDL), glucose, and creatinine. As creatinine was only assessed in a subsample of 2000 participants from RS-I-4 and RS-II-2, we carried forward creatinine levels from the nearest center visit if measurements at baseline were unavailable. Diabetes was defined as a fasting serum glucose level ≥ 7 mmol/L, a non-fasting glucose level ≥ 11.1 mmol/L, or the use of antidiabetic medication. Body mass index (BMI) was computed using the height (in cm) and weight (in kg) (kg/m2). *APOE*-ε4 carrier status was determined by polymerase chain reaction on coded DNA samples for RS-I and with a biallelic TaqMan assay (rs7412 and rs429358) for RS-II (Wenham et al., 1991). In 111 participants with missing *APOE* genotypes from this blood sampling, genotype was determined by genetic imputation (Illumina 610 K and 660 K chips; imputation with the Haplotype Reference Consortium reference panel (v1.0) with Minimac 3). History of clinical stroke was assessed at baseline visit and through continuous follow-up screening; details are described in the supplementary methods.

### Statistical analysis

Extreme NfL values were truncated at ± 3.5 standard deviations (*n* = 39). WMH volume and NfL were then natural log-transformed to obtain a normal distribution of the data. We subsequently standardized baseline NfL, cognition, and volumetric brain MRI measurements by dividing the difference between the individual value and the population mean by the population standard deviation. The presence of microbleeds and lacunes were modeled dichotomously, and microbleeds were additionally categorized as no microbleeds (0), one microbleed (1), or more than 2 microbleeds (2). Missing data ranged from 0.1% for hypertension to 9.3% for creatinine and were imputed 10 times with 20 iterations using chained equations (MICE R package v3.12.0) [29].

First, we determined the associations between plasma NfL levels and cognitive functioning, using linear mixed-effects models with random intercepts and slopes. We fitted a model under restricted maximum likelihood, with a diagonal covariance structure for the random effects, using follow-up time in years from baseline as the time variable. To investigate both the association between plasma NfL levels and cognitive function at baseline as well as whether NfL accelerates cognitive decline, plasma NfL and the interaction between plasma NfL and follow-up time were both integrated in the model. We additionally included a two-way interaction between follow-up time and age at baseline to account for possible slope differences depending on the baseline age. To control for confounding, we constructed two models. In the first model, we adjusted for age at baseline, age^2^, sex, assay batch number, and educational attainment. In the second model, we additionally adjusted for creatinine levels, cholesterol levels, smoking status, diabetes, body mass index, and hypertension.

Next, we determined associations between plasma NfL and neuroimaging markers, using similar models as those described above for cognition (excluding education), with further adjustment for time between blood sampling and MRI, total intracranial volume, volume of normal-appearing white matter (for all DTI analyses), tract-specific white matter volume (for DTI tracts analyses), and log-transformed tracts-specific WMH volume (for DTI tracts analyses). For the medial lemniscus tract, the varying field of view was also added to account for the fact that the cerebellum could not always be fully incorporated in the diffusion scan window. Cross-sectional analyses of categorical outcome measures were performed using logistic regression models for the presence of lacunar infarcts and microbleeds and multinomial logit models for the microbleed categories. Because of the aforementioned change in the DTI protocol during the study period, we ran separate linear regression models for the cross-sectional DTI analyses, both with the old and new DTI protocol scans (see also Supplementary Fig. 1).

We graphically presented the temporal course by plotting the predicted values for the average participant with mean, ± 1 SD log plasma NfL levels throughout follow-up. In sensitivity analyses, we (1) stratified by median age (70 years), (2) stratified by sex, (3) additionally adjusted imaging analyses for WMH to explore whether associations were driven by WMH, (4) explored the effect of APOE4 status through additional adjustment, (5) reran all analyses excluding participants with prior stroke or stroke during follow-up to gauge potential mediation effects, and (6) reran all analyses excluding outcome measurements with a value of 3.5SD higher or lower than the mean to assess the influence of potential outliers. We corrected the significance level (α level of 0.05) for multiple comparisons with the number of independent tests on the basis of the variance of the eigenvalues of the correlation matrix of all variables used in the main analysis. The following formula was used: Meff = $\frac{[{\sum }_{m=1}^{M}{\surd {\lambda }_{m}]}^{2}}{\sum_{m=1}^{M}{\lambda }_{m}}$, in which M is the number of variables, λ_m_ is the the eigenvalues of the correlation matrix, and Meff is the number of independent tests. This resulted in an Meff of 18.30 for the tract-specific DTI outcomes, an Meff of 8.77 for the global neuroimaging outcomes, and an Meff of 6.29 for the cognition outcomes. Using the Šidák formula (*α*-Šidák = 1 − (1 − α)^1/Meff^), this translated into a significance level of *p* < 0.008 for the cognitive outcomes, *p* < 0.006 for the global neuroimaging outcomes, and *p* < 0.003 for the tract-specific DTI analyses [30].

All analyses were conducted using R statistical software packages (version 4.0.3; packages mlogit, stats (lm, glm), nlme (lme)) [31].

## Results

Baseline population characteristics of all 4705 participants are shown in Table 1. Mean age at baseline was 71.9 years (SD: 7.3), and 57% were women. The subset of 975 participants who underwent brain MRI was on average younger, more often men, and—after adjustment for age and sex—had a lower BMI and less often diabetes mellitus. Plasma NfL levels were similar in the imaging subsample compared to the overall study population (Table 1).

**Table 1:** Characteristics of the study population

Characteristic	Overall sample(*N* = 4705)	Subset with MRI(*N* = 975)	*p*-value^*^
Age, years	71.9 ± 7.3	67.7 ± 6.4	< 0.001
Women	2688 (57.1%)	502 (51.5%)	0.001
Education			0.005
Lower (primary, unfinished secondary and lower vocational)	1831 (38.9%)	282 (28.9%)	
Further (secondary or intermediate vocational education)	2213 (47%)	507 (52%)	
Higher (higher vocational education or university)	661 (14%)	186 (19.1%)	
Neurofilament light chain, pg/ml (median, (IQR))	13.2 (9.9–18.1)	11.3 (9.0–14.8)	0.78
Total cholesterol, mmol/L	5.6 ± 1.0	5.7 ± 1.0	0.054
Creatinine, µmol/L	81.9 ± 19.8	82.6 ± 20.8	0.069
Hypertension	3697 (78.6%)	695 (71.1%)	0.13
Smoking			0.31
Never	1385 (29.4%)	277 (28.4%)	
Former	2603 (55.3%)	558 (57.2%)	
Current	717 (15.2%)	140 (14.4%)	
Diabetes mellitus	578 (12.3%)	88 (9.0%)	0.037
Body mass index, kg/m^2^	27.6 ± 4.1	27.4 ± 3.7	0.017
Apolipoprotein E ε4 carriers	1242 (26.4%)	252 (25.8%)	0.17
White matter hyperintensities, ml (median, (IQR))	NA	4.0(2.3–7.7)	
Lacunes, *n*	NA	70 (7.2%)	
Microbleeds, *n*			
1	NA	123 (12.6%)	
≥ 2	NA	90 (9.2%)	
Intracranial volume, ml	NA	1139.3 ± 112.9	
Total brain volume, ml	NA	923.4 ± 93.5	
Gray matter volume, ml	NA	523.8 ± 51.4	
Hippocampal volume, ml^a^	NA	7.6 ± 0.8	
Mean cortical thickness, mm^a^	NA	2.4 ± 0.1	
Mean cortical thickness, ADsig Mayo, mm^a^	NA	2.7 ± 0.1	
Mean cortical thickness, ADsig Harvard, mm^a^	NA	2.5 ± 0.1	
Normal-appearing white matter volume, ml	NA	392.4 ± 57.4	
Fractional anisotropy^a^	NA	0.36 ± 0.02	
Mean diffusivity^a^, 10^−3^mm^2^/s	NA	0.77 ± 0.04	
G-factor^a^	0.0 ± 1.0	0.4 ± 0.9	0.001
Purdue pegboared, total number of pins placed^a^	33.3 ± 5.1	35.0 ± 4.7	0.004
WLT immediate, number of words remembered^a^	6.8 ± 2.0	7.2 ± 2.0	0.26
WLT delayed, number of words remembered^a^	6.5 ± 2.7	7.1 ± 2.6	0.014
Word fluency test, number of animals^a^	20.7 ± 5.3	21.8 ± 5.0	0.16
Stroop reading test, seconds^a^	18.6 ± 4.4	17.9 ± 4.0	0.98
Stroop color naming test, seconds^a^	24.8 ± 5.8	23.5 ± 4.6	0.079
Stroop interference test, seconds^a^	60.8 ± 30.2	52.0 ± 22.8	0.054
Letter-digit substitution test, total correct digits^a^	27.0 ± 7.0	29.8 ± 6.4	< 0.001

Data are averaged over 10 imputations and presented as means (± standard deviation) for continuous variables and numbers with percentages for categorical variables, unless stated otherwise. *adjusted for age and sex when applicable, ^a^missing values: apolipoprotein E allele 4 carriership (143 for cognition analyses, 25 for MRI analyses), hippocampal volume (50 participants), cortical thickness (50 participants), cognition was available in a subset of 972 participants with MRI measurements, and DTI measurements (fractional anisotropy and mean diffusivity) were available in a subset of 924 participants

ADsig Mayo: surface area-weighted mean cortical thickness in entorhinal cortex, fusiform, inferior and middle temporal regions

ADsig Harvard: surface area-weighted mean cortical thickness in entorhinal cortex, parahippocampus, inferior parietal lobe, pars opercularis, pars orbitalis, pars triangularis, inferior temporal, temporal pole, precuneus, supramarginal gyrus, superior parietal, and superior frontal regions

*IQR* inter quartile range, *MRI* magnetic resonance imaging, *N* number of participants, *NA* not applicable, *WLT* word learning test

### Plasma NfL and cognitive function

**Fig. 2:**
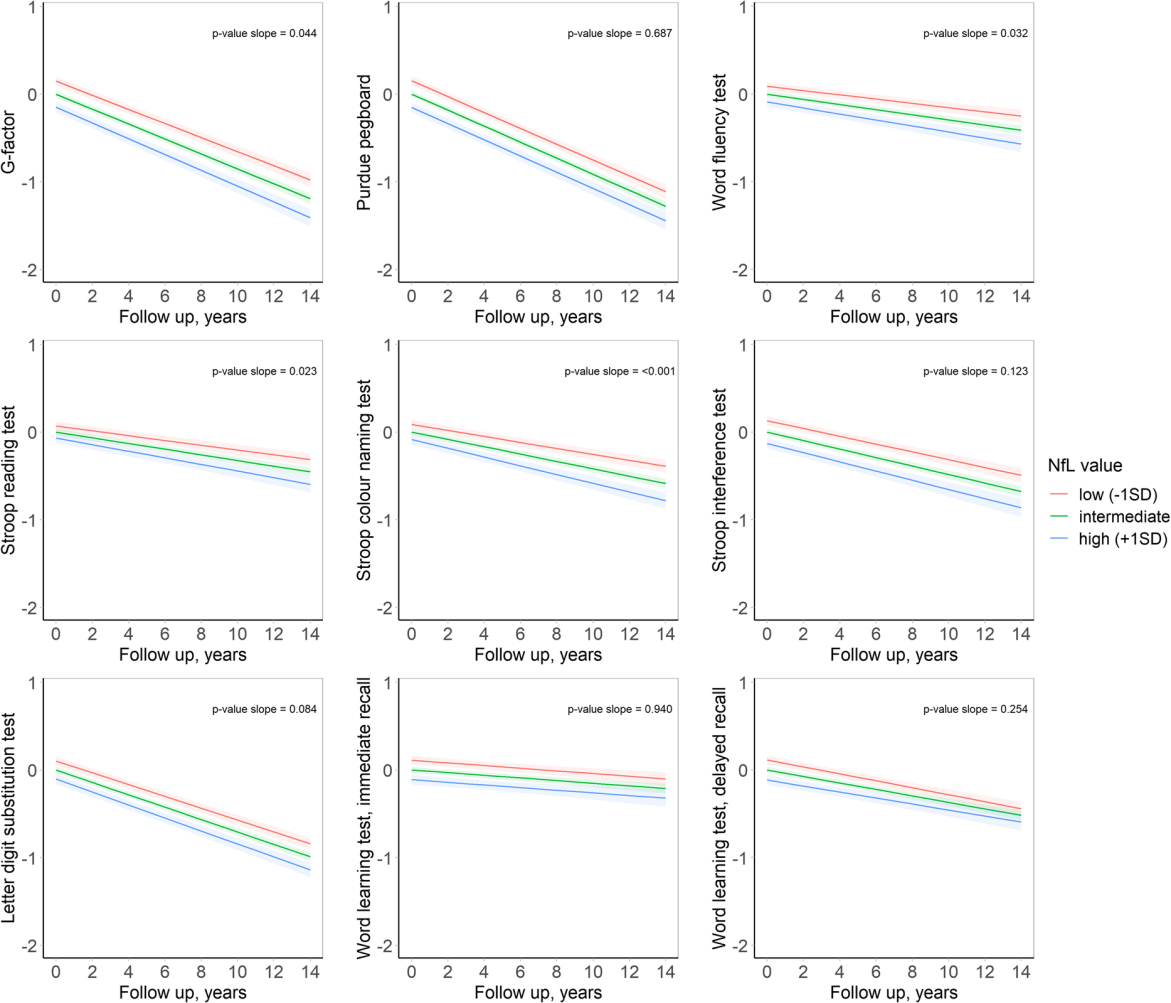
Trajectories of cognitive performance for different plasma neurofilament light chain values at baseline. Follow-up time in years is depicted on the *x*-axis, and the *y*-axis represents the standardized cognitive test scores. Predicted trajectories are plotted for an average person with a low (mean − 1SD), intermediate (mean), or high (mean + 1SD) NfL concentration. The trajectories of the stroop tests were multiplied with − 1 to orient all lines in the same direction. Abbreviations: *NfL* neurofilament light chain, *SD* standard deviation

Associations between plasma NfL and cognitive function are presented in Table 2 and Fig. 2. At baseline, higher plasma NfL was significantly associated with a worse performance on all cognitive tests, with similar effect estimates across cognitive domains (Table 2). Among 3045 participants with at least one repeated cognitive assessment, baseline NfL level was associated with a faster decline on the Stroop color naming task (*β* = 0.04 (0.02; 0.06), *p* < 0.001; Table 2). Similar, yet smaller, trends were observed for Stroop reading, verbal fluency, and the composite measure for global cognition, but these did not retain significance after adjustment for multiple testing (Table 2, Fig. 2).

**Table 2:** Association between plasma neurofilament light chain level and cognitive function

	Cross-sectional		Longitudinal	
	Mean difference (95%CI)	*p*-value	Slope difference (95%CI)	*p*-value
G-factor	− 0.12 (− 0.15; − 0.09)	< 0.001*	− 0.02 (− 0.04;0.00)	0.044
WLT immediate recall	− 0.10 (− 0.14; − 0.07)	< 0.001^*^	0.00 (− 0.02;0.02)	0.94
WLT delayed recall	− 0.11 (− 0.14; − 0.07)	< 0.001^*^	0.01 (− 0.01; 0.04)	0.25
Word fluency test	− 0.08 (− 0.12; − 0.05)	< 0.001^*^	− 0.02 (− 0.04; 0.00)	0.032
Stroop reading test	0.06 (0.02;0.10)	0.001^*^	0.02 (0.00;0.04)	0.023
Stroop color naming test	0.08 (0.04;0.12)	< 0.001^*^	0.04 (0.02;0.06)	< 0.001^*^
Stroop interference test	0.11 (0.08;0.15)	< 0.001^*^	0.02 (0.00; 0.04)	0.12
Letter-digit substitution test	− 0.09 (− 0.12; − 0.05)	< 0.001^*^	− 0.01 (− 0.03; 0.00)	0.08
Purdue pegboard	− 0.12 (− 0.15; − 0.09)	< 0.001^*^	0.00 (− 0.02; 0.02)	0.69

The table shows the association of plasma neurofilament light chain (NfL) at baseline with cognitive test scores at baseline (mean difference), and the association of baseline NfL with change in cognitive scores (slope difference). Higher scores reflect better performance on all tests, except for the Stroop tasks. The mean differences represent the difference in standardized cognitive test scores per standard deviation increase in log-transformed plasma NfL level at baseline. The slope differences represent the additional change in standardized cognitive test scores expressed per 5 years of follow-up. Models are adjusted for age at baseline, a non-linear term of age, sex, education, assay batch number, creatinine, cholesterol, smoking, diabetes mellitus, body mass index, and hypertension

Abbreviations: *WLT* word learning test, *CI* confidence interval

^*^significant at *p* < 0.008

Sensitivity analyses showed similar results for men and women (Supplementary Table 1). In the cross-sectional analyses, effect estimates were somewhat larger in older compared to younger participants across all cognitive tests, but no consistent age differences were observed regarding cognitive decline during follow-up (Supplementary Table 2). When excluding 209 participants with a history of clinical stroke at baseline and 105 participants with incident stroke, effect estimates in the cross-sectional as well as longitudinal analyses were slightly attenuated (Supplementary Table 3). Adjustment for the number of APOE4-ε4 alleles did not materially change the associations. Excluding outcome measurements with a value of 3.5 SD higher or lower than the mean in the Stroop tasks and g-factor models resulted in slightly reduced associations at baseline and over time (Supplementary Table 4).

### Plasma NfL and neuroimaging measures

**Fig. 3:**
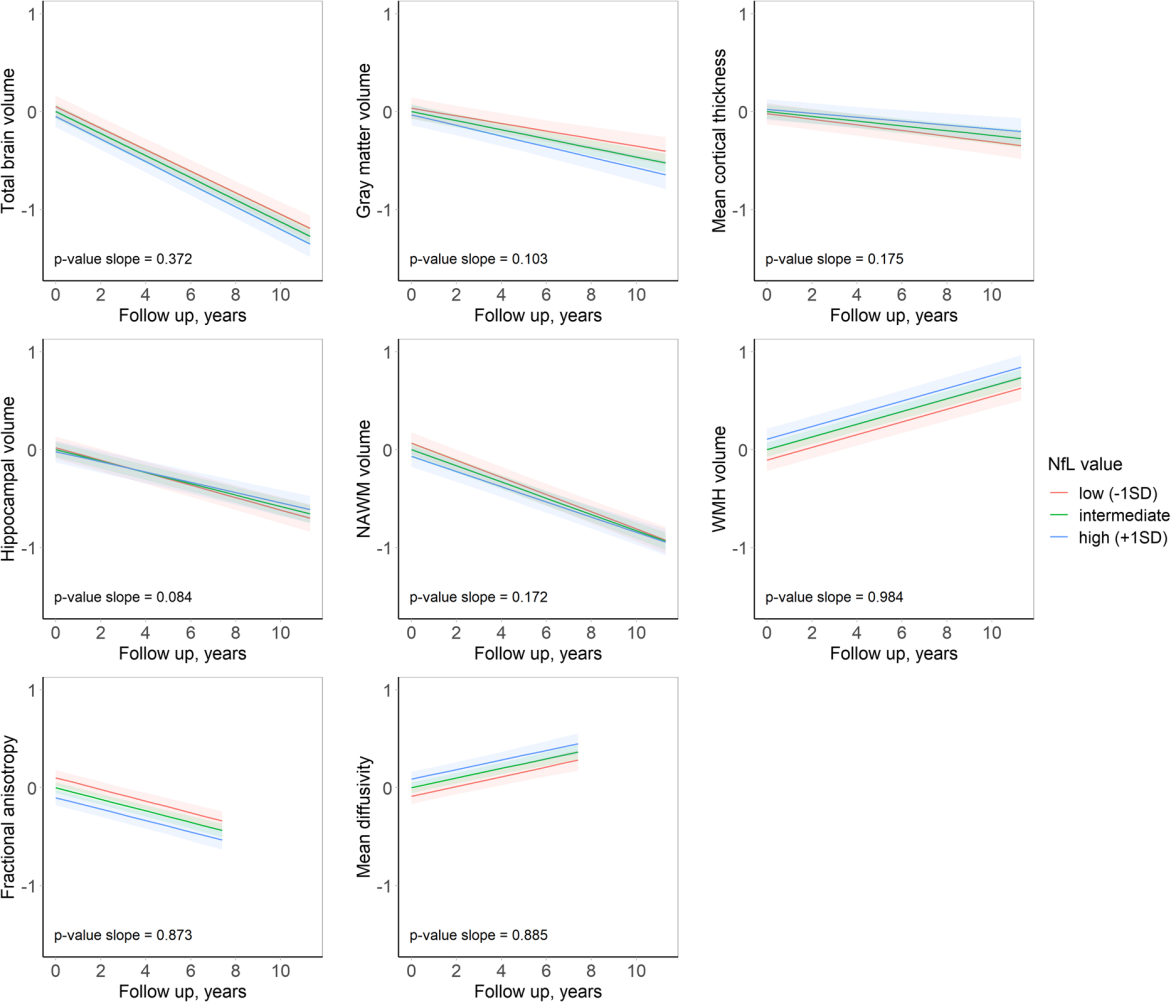
Trajectories of imaging markers for different plasma neurofilament light chain values at baseline. Follow-up time in years is depicted on the *x*-axis, and the *y*-axis represents the standardized imaging markers. Predicted trajectories are plotted for an average person with a low (mean − 1SD), intermediate (mean), or high (mean + 1SD) NfL concentration. Abbreviations: *WMH* white matter hyperintensities, *NAWM* normal-appearing white matter, *NfL* neurofilament light chain, *SD* standard deviation

Associations between plasma NfL and brain volumes, white matter integrity, and markers of cerebral small-vessel disease are presented in Table 3 and Fig. 3. At baseline, we observed no associations of NfL with total gray matter volume or cortical thickness, nor with volumes of the hippocampus or AD-specific regions in particular (Table 3, Fig. 3, Supplementary Fig. 2). In contrast, higher NfL levels were significantly associated with worse white matter integrity (MD: *β* = 0.12 (0.06; 0.19), *p* < 0.001), with similar trends for larger volume of WMH and the presence of lacunes but not cerebral microbleeds (Table 3, Fig. 3, Supplementary Fig. 2). Associations with white matter integrity were most profound for the association and projection tracts (Supplementary Table 5). Associations with global MD retained significance after further adjustment for WMH volume (*β* = 0.11; 95% CI: 0.05; 0.17, *p* < 0.001) and attenuated slightly when excluding MD measurements with a value of 3.5 SD higher or lower than the mean (*β* = 0.08; 95% CI: 0.01; 0.15, *p* = 0.023 Supplementary Table 4).

**Table 3:** Association between plasma neurofilament light chain level and neuroimaging markers

	Cross-sectional		Longitudinal	
	Mean differences (95%CI)	*p*-value	Slope differences (95%CI)	*p*-value
Total brain volume	− 0.02 (− 0.05; 0.01)	0.23	0.00 (− 0.02; 0.01)	0.37
Gray matter volume	− 0.02 (− 0.06; 0.03)	0.44	− 0.02 (− 0.05; 0.00)	0.10
Hippocampal volume	− 0.02 (− 0.08; 0.04)	0.61	0.02 (0.00; 0.05)	0.08
Mean cortical thickness	0.01 (− 0.06; 0.08)	0.80	0.02 (− 0.01; 0.05)	0.18
Mean cortical thickness Mayo AD signature ROI	− 0.02 (− 0.09; 0.06)	0.68	0.04 (0.00; 0.08)	0.044
Mean cortical thickness Harvard AD signature ROI	0.02 (− 0.06; 0.09)	0.67	0.02 (− 0.01; 0.05)	0.26
Normal-appearing white matter volume	− 0.04 (− 0.09; 0.01)	0.09	0.02 (− 0.01; 0.04)	0.17
White matter hyperintensities volume	0.09 (0.02; 0.16)	0.011	0.00 (− 0.02; 0.02)	0.98
Fractional anisotropy	− 0.07 (− 0.14; 0.00)	0.049	0.00 (− 0.03; 0.03)	0.87
Mean diffusivity	0.12 (0.06; 0.19)	< 0.001*	0.00 (− 0.04; 0.03)	0.89
	Odds ratio (95% CI)			
Lacunar infarcts presence	1.55 (1.13; 2.14)	0.007	NA	
Microbleeds presence (any vs. none)	0.95 (0.77; 1.18)	0.70	NA	
Microbleed factor (1 vs. 0)	0.86 (0.66; 1.13)	0.39	NA	
Microbleed factor (≥ 2 vs. 0)	1.07 (0.79; 1.44)	0.71	NA	

The table shows the association of plasma neurofilament light chain (NfL) at baseline with neuroimaging markers at baseline (mean difference/odds ratio) and the association of baseline NfL with change in neuroimaging markers (slope difference). The mean differences represent the difference in standardized neuroimaging per standard deviation increase in log-transformed plasma NfL level at baseline. The slope differences represent the additional change in standardized neuroimaging markers expressed per 5 years of follow-up. Models are adjusted for age, age^2^, sex, time between blood sampling and neuroimaging, intracranial volume, normal-appearing white matter (for DTI analyses only), assay batch number, creatinine, cholesterol, smoking, diabetes mellitus, body mass index, and hypertension

Abbreviations: *CI* confidence interval, *NA* not applicable

^*^significant at *p* < 0.006

Among 808 participants with at least one repeated MRI scan, plasma NfL levels were not significantly associated with change in any of the brain tissue gray or white matter parameters (Table 3, Fig. 3, Supplementary Fig. 2). Results for the various white matter tracts were consistent with those for global white matter integrity (Supplementary Table 5). A trend towards a *slower* decline in hippocampal volume and cortical thickness in AD signature regions (Mayo clinic definition) with higher NfL was not statistically significant when accounting for multiple testing and was less profound in the Harvard AD regions of interest (Table 3, Fig. 3).

In sex-stratified analyses, associations of NfL with baseline white matter volume, microstructural integrity, and cortical thickness were somewhat stronger in women than in men, whereas effect estimates were stronger in men for baseline WMH and gray matter volume, but neither interaction was statistically significant (Supplementary Table 6). The associations of NfL with a slower decline in mean cortical thickness and cortical thickness in AD signature regions (Mayo clinic definition) were more evident in women (Supplementary Table 6). Results did not differ by baseline age (Supplementary Table 7). The cross-sectional analyses between NfL and microstructural changes were similar for scans from the old DTI protocol compared to those from the new DTI protocol (Supplementary Table 8). When excluding 22 participants with a history of stroke before their baseline scan and 10 participants with incident stroke, effect estimates in the cross-sectional analyses attenuated slightly for WMH and lacunar infarcts (Supplementary Table 9), while estimates in the longitudinal analyses slightly increased for hippocampal volume and cortical thickness (Supplementary Table 9). Adjustment for the number of APOE4-ε4 alleles did not materially change the associations.

## Discussion

In this population-based study, higher plasma NfL levels were associated with worse cognitive performance and larger burden of primarily white matter pathology on brain MRI. Although participants with high NfL declined faster on repeated cognitive assessment over 10 years of follow-up, differences were relatively small and not accompanied by measurable changes in brain imaging markers of either white matter or gray matter pathology.

In various clinic-based studies, plasma NfL was robustly associated with cognitive decline in patients with MCI and AD but not in cognitively unimpaired individuals [9, 11, 12, 32]. In dementia-free individuals in less-selected community-dwelling and population-based samples, higher plasma NfL was generally found to be associated with worse cognitive performance and accelerated cognitive decline [4, 6, 8, 13–15]. The current study corroborates these findings in the largest population to date over a long follow-up period of 10 years. Although effect estimates were generally small, NfL was most strongly associated with changes in the Stroop reading and naming tasks and verbal fluency, which might suggest that NfL is most sensitive to changes in information processing speed. This was, however, not reflected in the association with LDST, possibly because this test additionally relies on other cognitive abilities which could be impacted in a later neurodegenerative disease stage. While our results suggest that plasma NfL may be used as a minimally invasive and easily obtainable biomarker of cognitive decline in dementia-free individuals from the general population, further study is needed to determine if the predictive value of NfL is sufficient to serve, for example, treatment selection or inclusion in clinical trials. Given the relatively small effect estimates in this cognitively healthy population, such an approach likely warrants a combination of plasma NfL with markers of neurodegeneration that capture other parts of the broad spectrum of pathology that underlies the dementia phenotype, notably plasma amyloid-b42 and phosphorylated tau [3, 4].

To address the biological definition of Alzheimer’s disease, including accumulation of amyloid (A) plaques, deposition of tau (T) tangles, and neuronal injury/neurodegeneration (N), the National Institute on Aging and Alzheimer’s Association proposed the ATN research framework [33]. The usefulness of NfL as a surrogate marker of neurodegenerative disease and its complementary value to other biomarkers within the ATN framework largely depend on the pathophysiological substrate of increases in plasma NfL concentration. The neuroimaging findings of the current study show that NfL is primarily associated with white matter pathology at baseline. Although this is consistent with the axonal origin of NfL and with previous research identifying NfL mainly in the cerebral white matter [34], prior studies on the biological underpinning of elevated NfL have been rather equivocal. Both in clinical and population setting, NfL in cognitively unimpaired participants has been linked to a degree of gray matter atrophy [13–15, 32, 35], although not necessarily with progressive gray matter atrophy over time [6, 14–16]. Differences between studies could relate to the stage of neurodegenerative disease participants are in, with axonal damage contributing to “upstream” neuronal loss later in the disease trajectory. The associations of NfL with white matter pathology—in the absence of gray matter atrophy—may thus reflect a population in an earlier disease stage, during which upstream neurodegenerative consequences of white matter pathology have not yet manifested. In support of this theory, the vast majority of the aforementioned studies did observe associations of plasma NfL with WMH [6, 13–15].

While we observed clear associations between NfL and white matter injury cross-sectionally, this did not translate into changes in white matter pathology during follow-up. These observations are in line with other studies [6, 13–15], neither of which found associations between plasma NfL and change in WMH volume over time. The discrepancy between cross-sectional and longitudinal results could imply reversed causality, in which changes in plasma NfL follow those in imaging markers. Alternatively, it could relate to the limited sensitivity of WMH burden for adequately reflecting the entire spectrum of white matter pathology. In two previous longitudinal studies among participants without dementia from the Mayo Clinic Study of Aging and the Betula study, baseline NfL did correlate to more rapid decline in the microstructure of the corpus callosum [15, 16]. Even though microstructural integrity using DTI is more sensitive to detect change in subclinical white matter pathology than WMH volume, we could not replicate these DTI findings over a longer follow-up in the current study. On the contrary, higher plasma NfL tended to predispose to a *slower* decline in AD signature regions, mostly in women. These findings could be due to true, unknown biological mechanisms, or represent a methodological artifact due, for instance, to attrition bias. Older and less healthy participants, who often have higher NfL values, are less likely to participate in follow-up examinations, which can lead to attenuated or reversed associations. The usefulness of NfL for monitoring disease severity, as well as treatment effects, warrants further longitudinal studies with repeated measurements of NfL, in parallel to repeated cognitive assessment and neuroimaging, and should further explore potential sex differences. Still, the apparent specificity of NfL to white matter pathology can steer its application, for example, towards trials aimed at the prevention of progression of cerebral small-vessel disease and lacunar stroke. We have previously shown that higher plasma NfL also increases the risk of clinical stroke [36], which may mediate at least some of the association between NfL and cognitive decline, as excluding participants with a clinical stroke in our study generally attenuated the effect estimates.

Although we believe our results are valid, several limitations should be taken into account. First, we lacked repeated measures of NfL to assess changes in plasma NfL levels concurrent with cognitive decline and changes in neuroimaging markers. Second, the time lag between measurement of plasma NfL and measures on MRI may have led to selection bias—and most likely an underestimation of the true effect estimate— as those with the highest NfL level and worst brain health might have died, declined to attend, or dropped out prior to the MRI study visit. Third, attrition may have hampered the ability to demonstrate an association between baseline NfL and changes in brain imaging markers. Fourth, our study population is predominantly White (97%), potentially limiting external generalizability. Fifth, the DTI protocol changed for the follow-up assessments, but mean differences in FA and MD were similar using linear regression models with the old DTI protocol data (2005 scans) and the new DTI protocol data (2008 scans), correcting for the time difference between plasma NfL and scan measurement. Strengths of this study include the large sample size, its population-based design, the correction for many potential confounders, and the long follow-up of 10 years.

In conclusion, higher plasma NfL levels are associated with cognitive decline and larger burden of primarily white matter pathology in this community-dwelling population aged 55 years and over. These findings further establish NfL as a marker of axonal damage in the preclinical phase of neurodegenerative disease.

### Supplementary Information

Below is the link to the electronic supplementary material.

Supplementary file1 (DOCX 363 KB)

## Data Availability

Data can be obtained upon request. Requests should be directed towards the management team of the Rotterdam Study (secretariat.epi@erasmusmc.nl), which has a protocol for approving data requests. Because of restrictions based on privacy regulations and informed consent of the participants, data cannot be made freely available in a public repository. The Rotterdam Study has been approved by the Medical Ethics Committee of the Erasmus MC (registration number MEC 02.1015) and by the Dutch Ministry of Health, Welfare and Sport (Population Screening Act WBO, license number 1071272-159521-PG). The Rotterdam Study has been entered into the Netherlands National Trial Register (NTR; www.trialregister.nl) and into the WHO International Clinical Trials Registry Platform (ICTRP; www.who.int/ictrp/network/primary/en/) under shared catalogue number NTR6831. All participants provided written informed consent to participate in the study and to have their information obtained from treating physicians.

## References

[1] Jack CR (2013). Tracking pathophysiological processes in Alzheimer’s disease: an updated hypothetical model of dynamic biomarkers. Lancet Neurol.

[2] Khalil M (2018). Neurofilaments as biomarkers in neurological disorders. Nat Rev Neurol.

[3] de Wolf F (2020). Plasma tau, neurofilament light chain and amyloid-beta levels and risk of dementia; a population-based cohort study. Brain.

[4] Cullen NC (2021). Plasma biomarkers of Alzheimer’s disease improve prediction of cognitive decline in cognitively unimpaired elderly populations. Nat Commun.

[5] Mattsson N (2017). Association of plasma neurofilament light with neurodegeneration in patients with Alzheimer disease. JAMA Neurol.

[6] Khalil M (2020). Serum neurofilament light levels in normal aging and their association with morphologic brain changes. Nat Commun.

[7] Mielke MM (2019). Plasma and CSF neurofilament light: relation to longitudinal neuroimaging and cognitive measures. Neurology.

[8] Chatterjee P (2018). Association of plasma neurofilament light chain with neocortical amyloid-beta load and cognitive performance in cognitively normal elderly participants. J Alzheimers Dis.

[9] Pereira JB (2021). Untangling the association of amyloid-beta and tau with synaptic and axonal loss in Alzheimer’s disease. Brain.

[10] Sanchez-Valle R (2018). Serum neurofilament light levels correlate with severity measures and neurodegeneration markers in autosomal dominant Alzheimer’s disease. Alzheimers Res Ther.

[11] Osborn KE (2019). Cerebrospinal fluid and plasma neurofilament light relate to abnormal cognition. Alzheimers Dement (Amst).

[12] He L (2021). Plasma neurofilament light chain is associated with cognitive decline in non-dementia older adults. Sci Rep.

[13] Rubsamen N (2021). Serum neurofilament light and tau as prognostic markers for all-cause mortality in the elderly general population-an analysis from the MEMO study. BMC Med.

[14] Rajan KB (2020). Remote blood biomarkers of longitudinal cognitive outcomes in a population study. Ann Neurol.

[15] Marks JD (2021). Comparison of plasma neurofilament light and total tau as neurodegeneration markers: associations with cognitive and neuroimaging outcomes. Alzheimers Res Ther.

[16] Nyberg L (2020). Elevated plasma neurofilament light in aging reflects brain white-matter alterations but does not predict cognitive decline or Alzheimer’s disease. Alzheimers Dement (Amst).

[17] Ikram MA (2020). Objectives, design and main findings until 2020 from the Rotterdam Study. Eur J Epidemiol..

[18] Ikram MA (2015). The Rotterdam scan study: design update 2016 and main findings. Eur J Epidemiol.

[19] Rissin DM (2010). Single-molecule enzyme-linked immunosorbent assay detects serum proteins at subfemtomolar concentrations. Nat Biotechnol.

[20] Rohrer JD (2016). Serum neurofilament light chain protein is a measure of disease intensity in frontotemporal dementia. Neurology.

[21] Vrooman HA (2007). Multi-spectral brain tissue segmentation using automatically trained k-nearest-neighbor classification. Neuroimage.

[22] de Boer R (2009). White matter lesion extension to automatic brain tissue segmentation on MRI. Neuroimage.

[23] Jack CR (2015). Different definitions of neurodegeneration produce similar amyloid/neurodegeneration biomarker group findings. Brain.

[24] Dickerson BC (2011). Alzheimer-signature MRI biomarker predicts AD dementia in cognitively normal adults. Neurology.

[25] Koppelmans V (2014). Global and focal white matter integrity in breast cancer survivors 20 years after adjuvant chemotherapy. Hum Brain Mapp.

[26] de Groot M (2015). Tract-specific white matter degeneration in aging: the Rotterdam Study. Alzheimers Dement.

[27] Hoogendam YY (2014). Patterns of cognitive function in aging: the Rotterdam Study. Eur J Epidemiol.

[28] Committee G (2003). European society of hypertension–European society of cardiology guidelines for the management of arterial hypertension*. J Hypertens.

[29] van Buuren S, Groothuis-Oudshoorn K (2011). Mice: multivariate imputation by chained equations in R. J Stat Softw.

[30] Galwey NW (2009). A new measure of the effective number of tests, a practical tool for comparing families of non-independent significance tests. Genet Epidemiol.

[31] Team RC (2019). R: A language and environment for statistical computing.

[32] Moscoso A (2021). Longitudinal associations of blood phosphorylated tau181 and neurofilament light chain with neurodegeneration in Alzheimer disease. JAMA Neurol.

[33] Jack CR (2018). NIA-AA research framework: toward a biological definition of Alzheimer’s disease. Alzheimers Dement:J Alzheimers Assoc.

[34] Sjolin K (2022). Distribution of five clinically important neuroglial proteins in the human brain. Mol Brain.

[35] Benedet AL (2020). Stage-specific links between plasma neurofilament light and imaging biomarkers of Alzheimer’s disease. Brain.

[36] Heshmatollah A (2022). Plasma β-amyloid, total-tau, and neurofilament light chain levels and the risk of stroke: a prospective population-based study. Neurology.

