# Supplementary material for: Plasma neurofilament light chain in relation to 10-year change in cognition and neuroimaging markers: a population-based study
Source: GeroScience. 2023 Aug 3;46(1):57–70. doi: 10.1007/s11357-023-00876-5 (PMC10828339; doi:10.1007/s11357-023-00876-5)
Supplement: Supplementary file 1 — Supplementary file1 (DOCX 363 KB) [file 11357_2023_876_MOESM1_ESM.docx]

**Supplements**

**Methods and Materials**

**Dementia assessment**

Participants were screened for dementia at each visit with the Mini-mental State Examination and the Geriatric Mental State Schedule organic level. Those with a Mini-Mental State Examination score <26 or Geriatric Mental Schedule score >0 underwent further investigation and informant interview, including the Cambridge Examination for Mental Disorders of the Elderly. In addition, the entire cohort was continuously under surveillance for dementia through electronic linkage of the study database with medical records from general practitioners and the regional institute for outpatient mental health care. A consensus panel led by a consultant neurologist established the final diagnosis according to standard criteria for dementia (Diagnostic and Statistical Manual of Mental Disorder, Third Edition-Revised: DSM-III-R).

**Stroke assessment**

Stroke was defined according to the WHO criteria as a syndrome of rapidly developing clinical signs of focal or global disturbance

of cerebral function, with symptoms lasting 24 hours or longer or leading to death, with no apparent cause other than of vascular

origin. [1] History of stroke was determined during baseline interview and verified using medical records from general practitioners. Participants were continuously monitored for incident stroke by automated linkage of general practitioners’ and nursing home physicians’ medical records with the study database, similar to dementia assessment, and information from hospital records was obtained. Research physicians reviewed all information on potential strokes, and cases were verified by two experienced stroke neurologists in a consensus panel, corresponding with ICD-10 codes I61, I63 and I64.

**MRI acquisition and processing**

Multi-sequence MR imaging was performed on a 1.5 tesla MRI scanner (GE Signa Excite). A quality assurance protocol ensured that the system remained unchanged (i.e. no major updates or upgrades of scanner hard- and software) during the period of inclusion. The imaging protocol and sequence details have been described extensively elsewhere. [2] In brief, the structural imaging protocol consisted of a T1- weighted sequence (T1w, TR = 13.8 ms, TE = 2.80 ms, TI = 400 ms, 96 slices of 1.6 mm), a proton density-weighted sequence (PD TR = 21,300 ms, TE = 17.3, 90 slices of 1.6 mm), and a T2-weighted fluid-attenuated inversion recovery (FLAIR) sequence (TR = 8000 ms, TE = 120 ms, TI = 2000 ms, 64 slices of 2.5 mm).

We automatically segmented scans using an in-house developed method into grey matter (GM), white matter (WM), cerebrospinal fluid (CSF) and background tissue.[3] In brief, k-Nearest-Neighbor classification was automated by non-rigidly registering MR data with a tissue probability atlas to automatically select training samples, followed by a post-processing step to keep the most reliable samples. [3] We classified white matter hyperintensities (WMH) as a separate tissue class using a post-processing WMH segmentation approach, based on the FLAIR image and segmented brain tissues.[4] In brief, WMH are segmented using the brain tissue segmentation and a FLAIR scan. A WMH threshold for the FLAIR image is determined by using the FLAIR intensity histogram within a GM mask, obtained from the GM segmentation. It is followed by a simple post-processing step to ensure that the lesions found are within the white matter. This is accomplished by thresholding the WM fraction of neighboring voxels for every lesion. [4] Intracranial volume (ICV) (excluding the cerebellum and surrounding CSF) was estimated by summing total grey and white matter and CSF volumes. [3] Cortical infarcts were rated on structural sequences, and we classified them as cortical infarcts in case of involvement of cortical grey matter.[5]

We performed a single shot, diffusion-weighted spin echo echo-planar imaging sequence for diffusion-MRI (Old protocol: TR = 8000 ms, TE = 68.7 ms, axial field of view = 210 x 210 mm, 36 contiguous slices of 3.5 mm; New protocol: TR = 8575 ms, TE = 82.6 ms, axial field of view = 210 × 210 mm, 35 contiguous slices of 3.5 mm). Maximum b-value was 1000 s/mm2 in 25 non-collinear directions and three volumes were acquired without diffusion weighting (b-value = 0 s/mm2 ) in the new DTI protocol and one volume without diffusion in de the old DTI protocol. A standardized pipeline was used to preprocess the diffusion data (including correction for motion and eddy currents).[6] Voxelwise analysis of the diffusion data was performed with tract-based spatial statistics (TBSS).[7] TBSS registers all FA images to standard space, and then creates a study specific skeleton of the major white matter tracts (threshold for skeleton FA ≥ 0.2). To overcome residual misalignment after the registration, TBSS projects the maximum FA values in a line perpendicular to the tract onto the skeleton for each individual. The projection results in a series of skeletonized images that can be analyzed voxelwise. Additionally, the projection also maps DTI parameters other than the FA on the skeleton, allowing voxelwise analyses of MD. Global FA/MD values were computed in two ways: (a) We averaged the values of all voxels within the tract-based spatial statistics skeleton. (b) We averaged FA/MD inside the cerebral NAWM for each subject. First, for each subject, the T1 scan was coregistered to the FA, using a 12 degrees-of-freedom affine registration, as implemented by FLIRT [FMRIB’s Linear Image Registration Tool] [8]. The registration was used to resample the NAWM tissue segmentation in the space of the diffusion metrics. Next, diffusion metrics were averaged inside the NAWM tissue class in those regions where FA ≥ 0.2. [9]

The diffusion data were also used to segment white matter tracts using a diffusion tractography approach described previously.[10] The tract-specific analysis was performed incorporating all voxels of the tract anatomy, both normal-appearing white matter voxels and voxels containing WMHs. Tractography was performed in native space, using standard space seed, target, stop, and exclusion masks as described previously[10]. Tractography was performed with PROBTRACKX, a Bayesian framework for white matter tractography, available in FSL (version 4.1.4). Protocols for identifying 15 white matter tracts were defined as described previously and were made available as the autoPTX plugin for FSL (version 0.1.1). The amount of seed points was variable across tracts to achieve a robust sampling of all tracts investigated. The ball and stick diffusion model (BedpostX) estimation and tractography algorithm were run with default settings. We segmented 15 different white matter tracts (12 bilateral, 3 singular) and obtained mean FA and MD in these tracts, with subsequent combination of left and right measures[10]. Subsequently, we computed z-scores (subtracting the mean and dividing by the standard deviation) for the tract-specific parameters to facilitate comparison of associations. We combined the tissue and tract segmentations to obtain tract-specific white matter volumes and tract-specific WMH volumes (natural-log transformed to account for their skewed distribution). The 15 tracts were categorized according to presumed functional groups into brainstem tracts (middle cerebellar peduncle, medial lemniscus), projection tracts (corticospinal tract, anterior thalamic radiation, superior thalamic radiation, posterior thalamic radiation), association tracts (superior longitudinal fasciculus, inferior longitudinal fasciculus, inferior fronto-occipital fasciculus, uncinate fasciculus), limbic system tracts (cingulate gyrus part of cingulum, parahippocampal part of cingulum, fornix) and callosal tracts (forceps major, forceps minor). [10]


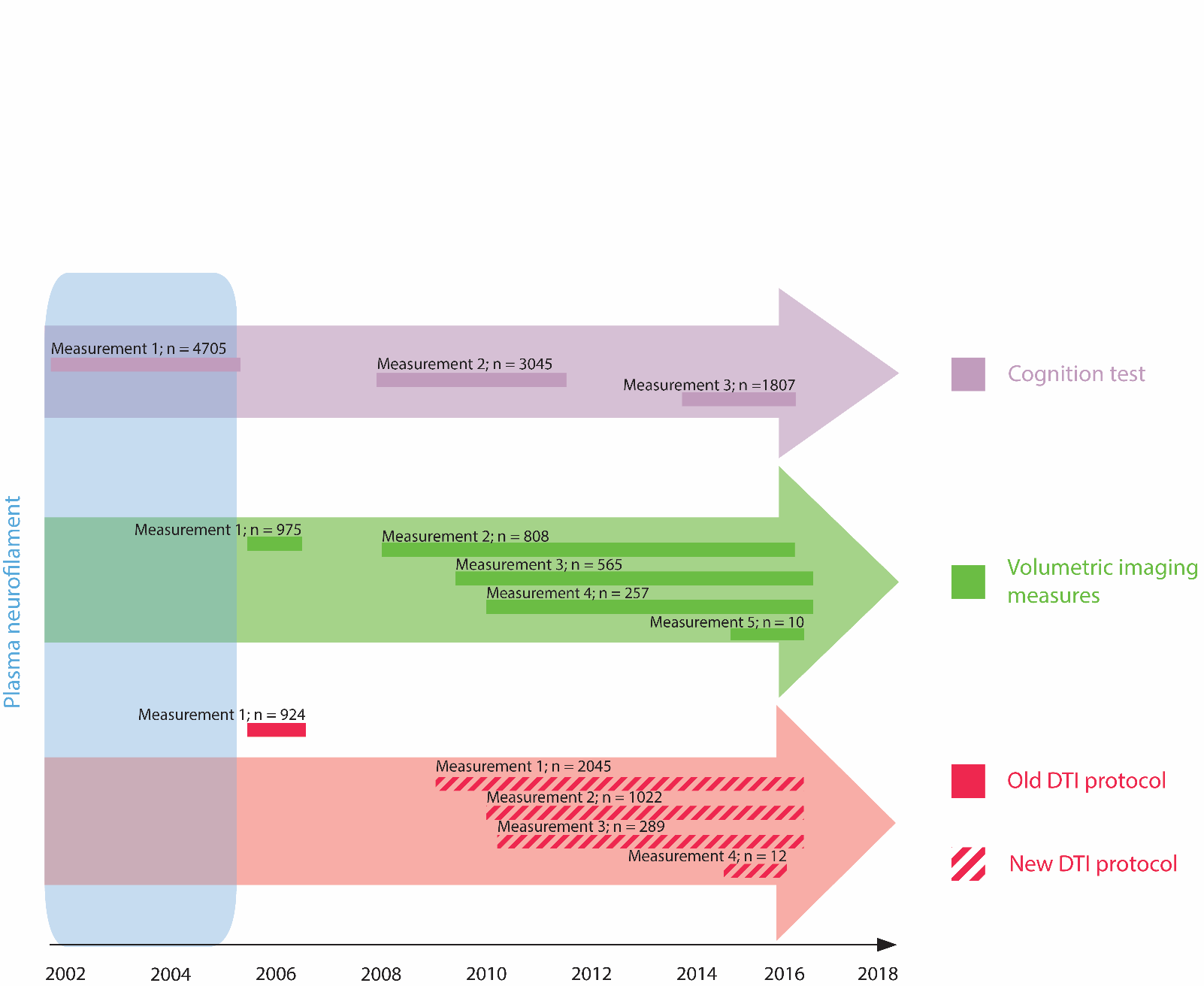


**Supplementary Fig. 1 Study overview** Plasma neurofilament light chain and cognition were assessed at baseline (2002-2005), and MRI was implemented in 2005 in the core protocol of the Rotterdam Study. The scanner and imaging protocols were kept identical throughout the study duration, except for a change in the DTI sequence (red squares). To assure comparability of longitudinal measures, only DTI data acquired with the newest protocol (red striped squares) were included in the longitudinal DTI analyses. DTI data acquired with the old protocol (red solid square) were used for cross-sectional analyses. DTI = diffusion tensor imaging.


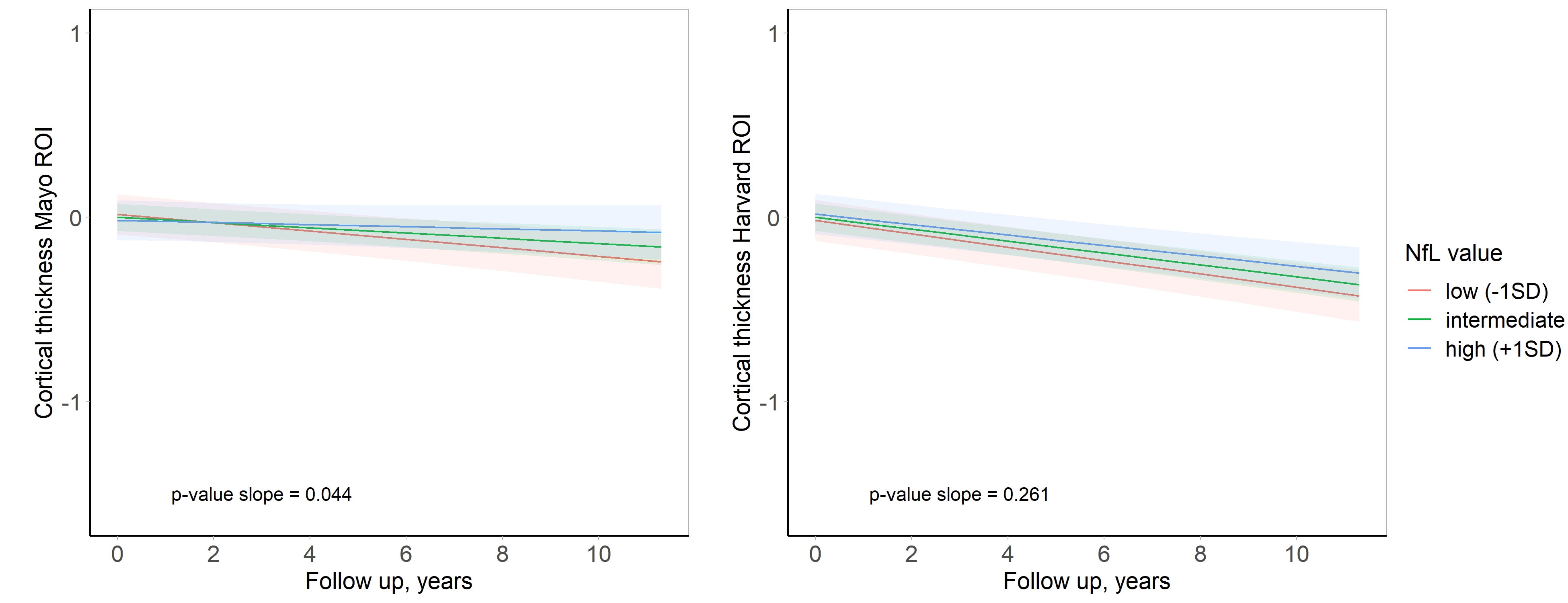


**Supplementary Fig.2 Trajectories of cortical thickness signatures for different plasma neurofilament light chain values at baseline** Follow-up time in years is depicted on the x-axis, the y-axis represents the standardized imaging markers. Predicted trajectories are plotted for an average person with a low (mean -1SD), intermediate (mean) or high (mean +1SD) NfL concentration. Abbreviations: NfL = neurofilament light chain, ROI = region of interest, SD = standard deviation.

**Supplementary Table 1**

|  | **Men** | **Women** |  |
| --- | --- | --- | --- |
|  | Mean differences (95%CI) | Mean differences (95%CI) | p-value for interaction |
| G-factor | -0.11(-0.16; -0.06) | -0.13(-0.18; -0.09) | 0.66 |
| WLT immediate recall | -0.12(-0.17; -0.07) | -0.09(-0.14; -0.04) | 0.64 |
| WLT delayed recall | -0.11(-0.16; -0.06) | -0.10(-0.15; -0.05) | 0.92 |
| Word fluency test | -0.06(-0.12; -0.01) | -0.10(-0.14; -0.05) | 0.97 |
| Stroop reading test | 0.07(0.02; 0.13) | 0.06(0.01; 0.11) | 0.80 |
| Stroop colour naming test | 0.07(0.02; 0.13) | 0.08(0.03; 0.13) | 0.36 |
| Stroop interference test | 0.10(0.05; 0.15) | 0.13(0.08; 0.18) | 0.27 |
| Letter-digit substitution test | -0.08(-0.13; -0.04) | -0.09(-0.14; -0.05) | 0.36 |
| Purdue pegboard | -0.13(-0.18; -0.08) | -0.12(-0.16; -0.08) | 0.27 |
|  |  |  |  |
|  | Slope differences (95% CI) | Slope differences (95% CI) | p-value for interaction |
| G-factor | -0.03(-0.06; 0.00) | -0.01(-0.04; 0.01) | 0.74 |
| WLT immediate recall | 0.01(-0.02; 0.04) | -0.01(-0.04; 0.02) | 0.30 |
| WLT delayed recall | 0.00(-0.03; 0.04) | 0.02(-0.01; 0.05) | 0.83 |
| Word fluency test | -0.03(-0.07; 0.00) | -0.02(-0.04; 0.01) | 0.47 |
| Stroop reading test | 0.02(-0.01; 0.05) | 0.02(-0.01; 0.05) | 0.36 |
| Stroop colour naming test | 0.05(0.01; 0.08) | 0.03(0.00; 0.05) | 0.60 |
| Stroop interference test | 0.00(-0.03; 0.03) | 0.03(0.00; 0.06) | 0.40 |
| Letter-digit substitution test | -0.02(-0.05; 0.00) | -0.01(-0.03; 0.02) | 0.35 |
| Purdue pegboard | -0.02(-0.05; 0.01) | 0.01(-0.02; 0.03) | 0.26 |
| The table shows the association of plasma neurofilament light chain (NfL) at baseline with cognitive test scores at baseline (mean difference), and the association of baseline NfL with change in cognitive scores (slope difference) in men and women separately. Higher scores reflect better performance on all tests, except for the Stroop tasks. The mean differences represent the difference in standardized cognitive test scores per standard deviation increase in log transformed plasma NfL level at baseline. The slope differences represent the additional change in standardized cognitive test scores expressed per 5 years of follow-up. Models are adjusted for age at baseline, a non-linear term of age, education, assay batch number, creatinine, cholesterol, smoking, diabetes mellitus, body mass index and hypertension. WLT = word learning test, CI = confidence interval. | | | |

**Supplementary Table 2**

|  | **Below 70 years** | **Above 70 years** |  |
| --- | --- | --- | --- |
|  | Mean differences (95%CI) | Mean differences (95%CI) | p-value for interaction |
| G-factor | -0.09(-0.14; -0.04) | -0.14(-0.19; -0.10) | 0.11 |
| WLT immediate recall | -0.06(-0.12; 0.00) | -0.14(-0.18; -0.09) | 0.06 |
| WLT delayed recall | -0.06(-0.12; -0.01) | -0.14(-0.19; -0.09) | 0.06 |
| Word fluency test | -0.03(-0.09; 0.02) | -0.12(-0.16; -0.07) | 0.04 |
| Stroop reading test | 0.05(0.00; 0.10) | 0.07(0.02; 0.13) | 0.31 |
| Stroop colour naming test | 0.05(0.00; 0.11) | 0.10(0.04; 0.15) | 0.18 |
| Stroop interference test | 0.06(0.02; 0.10) | 0.15(0.10; 0.21) | 0.007^*^ |
| Letter-digit substitution test | -0.07(-0.12; -0.02) | -0.10(-0.14; -0.06) | 0.32 |
| Purdue pegboard | -0.15(-0.20; -0.10) | -0.10(-0.15; -0.06) | 0.36 |
|  |  |  |  |
|  | Slope differences (95% CI) | Slope differences (95% CI) | p-value for interaction |
| G-factor | -0.02(-0.04; 0.01) | -0.03(-0.06; 0.01) | 1.00 |
| WLT immediate recall | -0.02(-0.05; 0.01) | 0.03(-0.01; 0.07) | 0.002^*^ |
| WLT delayed recall | 0.00(-0.02; 0.03) | 0.02(-0.02; 0.06) | 0.12 |
| Word fluency test | -0.03(-0.06; 0.00) | -0.02(-0.06; 0.01) | 0.69 |
| Stroop reading test | 0.02(0.00; 0.04) | 0.04(0.00; 0.07) | 0.72 |
| Stroop colour naming test | 0.04(0.01; 0.06) | 0.04(0.01; 0.08) | 0.87 |
| Stroop interference test | 0.03(0.00; 0.05) | 0.01(-0.04; 0.05) | 0.29 |
| Letter-digit substitution test | -0.01(-0.03; 0.01) | -0.02(-0.05; 0.01) | 0.91 |
| Purdue pegboard | 0.01(-0.02; 0.03) | -0.02(-0.05; 0.02) | 0.45 |
| The table shows the association of plasma neurofilament light chain (NfL) at baseline with cognitive test scores at baseline (mean difference), and the association of baseline NfL with change in cognitive scores (slope difference) stratified on age at baseline. Higher scores reflect better performance on all tests, except for the Stroop tasks. The mean differences represent the difference in standardized cognitive test scores per standard deviation increase in log transformed plasma NfL level at baseline. The slope differences represent the additional change in standardized cognitive test scores expressed per 5 years of follow-up. Models are adjusted for baseline age, a non-linear term of age, education, assay batch number, creatinine, cholesterol, smoking, diabetes mellitus, body mass index and hypertension. WLT = word learning test, CI = confidence interval. ^*^ =significant at p<0.008 | | | |

**Supplementary Table 3. Association between plasma neurofilament light chain level and cognitive function in persons without a clinical stroke**

|  | **Cross-sectional** |  | **Longitudinal** |  |
| --- | --- | --- | --- | --- |
|  | Mean difference (95%CI) | p-value | Slope difference (95%CI) | p-value |
| G-factor | -0.11(-0.14;-0.07) | <0.001* | -0.01(-0.03;0.01) | 0.19 |
| WLT immediate recall | -0.10(-0.14;-0.06) | <0.001* | 0.00(-0.02;0.03) | 0.86 |
| WLT delayed recall | -0.10(-0.14;-0.07) | <0.001* | 0.02(-0.01;0.04) | 0.16 |
| Word fluency test | -0.07(-0.11;-0.03) | <0.001* | -0.02(-0.04;0.00) | 0.08 |
| Stroop reading test | 0.05(0.01;0.08) | 0.017 | 0.02(0.00;0.04) | 0.06 |
| Stroop colour naming test | 0.07(0.03;0.10) | 0.001* | 0.02(0.00;0.04) | 0.021 |
| Stroop interference test | 0.10(0.07;0.14) | <0.001* | 0.00(-0.02;0.03) | 0.68 |
| Letter-digit substitution test | -0.07(-0.11;-0.04) | <0.001* | -0.01(-0.02;0.01) | 0.32 |
| Purdue pegboard | -0.11(-0.15;-0.08) | <0.001* | -0.01(-0.03;0.01) | 0.56 |
| The table shows the association of plasma neurofilament light chain (NfL) at baseline with cognitive test scores at baseline (mean difference), and the association of baseline NfL with change in cognitive scores (slope difference), excluding participants with a clinical stroke before their baseline cognitive assessment or during follow-up. Higher scores reflect better performance on all tests, except for the Stroop tasks. The mean differences represent the difference in standardized cognitive test scores per standard deviation increase in log transformed plasma NfL level at baseline. The slope differences represent the additional change in standardized cognitive test scores expressed per 5 years of follow-up. Models are adjusted for age at baseline, a non-linear term of age, sex, education, assay batch number, creatinine, cholesterol, smoking, diabetes mellitus, body mass index and hypertension. WLT = word learning test, CI = confidence interval. ^*^ =significant at p<0.008 | | | | |

**Supplementary Table 4. –Comparisons of the association between plasma NfL and measures of cognition and brain imaging in a dataset with and without outliers.**

|  | **Cross-sectional** |  | **Longitudinal** |  |
| --- | --- | --- | --- | --- |
| **Dataset without outliers** | Mean difference (95%CI) | p-value | Slope difference (95%CI) | p-value |
| Mean diffusivity, z-score | 0.08(0.01;0.15) | 0.023 | -0.01(-0.04;0.02) | 0.61 |
| Stroop reading, z-score | 0.05(0.01;0.08) | 0.005 | 0.02(0.00;0.03) | 0.07 |
| Stroop color naming, z-score | 0.06(0.03;0.09) | <0.001 | 0.03(0.01;0.04) | <0.001 |
| Stroop interference z-score | 0.08(0.06;0.11) | <0.001 | 0.00(-0.02;0.01) | 0.73 |
| Gfactor | -0.11(-0.14;-0.08) | <0.001 | -0.02(-0.03;0.00) | 0.07 |
|  |  |  |  |  |
| **Original dataset** |  |  |  |  |
| Mean diffusivity, z-score | 0.12(0.06; 0.19) | <0.001 | 0.00(-0.04;0.03) | 0.89 |
| Stroop reading z-score | 0.06(0.02;0.10) | 0.001 | 0.02(0.00;0.04) | 0.023 |
| Stroop color naming z-score | 0.08(0.04;0.12) | <0.001 | 0.04(0.02;0.06) | <0.001 |
| Stroop interference z-score | 0.11(0.08;0.15) | <0.001 | 0.02(0.00;0.04) | 0.123 |
| Gfactor | -0.12(-0.15;-0.09) | <0.001 | -0.02(-0.04;0.00) | 0.044 |
| The table shows the association of plasma neurofilament light chain (NfL) at baseline with cognitive test scores and neuroimaging markers at baseline (mean difference), and the association of baseline NfL with change in cognitive scores and neuroimaging markers(slope difference). Higher scores reflect better scores, except for the Stroop tasks. The mean differences represent the difference in standardized cognitive test scores per standard deviation increase in log transformed plasma NfL level at baseline. The slope differences represent the additional change in standardized cognitive test scores expressed per 5 years of follow-up. Models are adjusted for age at baseline, a non-linear term of age, sex, education (for the cognition tests), assay batch number, creatinine, cholesterol, smoking, diabetes mellitus, body mass index and hypertension. Individuals with an outcome value of 3.5SD higher or lower than the mean were identified as outliers and removed from the dataset without outliers to investigate their effects on the associations with plasma NfL. | | | | |

**Supplementary Table 5. Association between plasma neurofilament light chain level and DTI tract white matter integrity**

|  | **Fractional anisotropy** | | | | **Mean diffusivity** | | | |
| --- | --- | --- | --- | --- | --- | --- | --- | --- |
|  | **Cross-sectional** |  | **Longitudinal** |  | **Cross-sectional** |  | **Longitudinal** |  |
|  | Mean differences (95%CI) | p-value | Slope differences (95%CI) | p-value | Mean differences (95%CI) | p-value | Slope differences (95%CI) | p-value |
| **Brainstem tracts** |  |  |  |  |  |  |  |  |
| Middle cerebellar peduncle | -0.01(-0.06;0.04) | 0.63 | -0.05(-0.09;0.00) | 0.06 | 0.01(-0.04;0.05) | 0.79 | 0.03(-0.02;0.09) | 0.24 |
| Medial Lemniscus | -0.01(-0.06;0.03) | 0.53 | -0.02(-0.05;0.02) | 0.39 | 0.01(-0.04;0.06) | 0.70 | 0.03(-0.03;0.09) | 0.32 |
| **Projection Tracts** |  |  |  |  |  |  |  |  |
| Cortico-spinal tract | 0.01(-0.04;0.06) | 0.75 | 0.01(-0.02;0.05) | 0.50 | 0.09(0.04;0.13) | <0.001^*^ | -0.01(-0.06;0.03) | 0.50 |
| Anterior thalamic radiation | -0.08(-0.12;-0.04) | <0.001^*^ | 0.04(0.01;0.07) | 0.008 | 0.07(0.04;0.11) | <0.001^*^ | -0.02(-0.05;0.00) | 0.07 |
| superior thalamic radiation | -0.04(-0.09;0.01) | 0.14 | 0.00(-0.03;0.03) | 0.80 | 0.07(0.03;0.11) | 0.002^*^ | -0.01(-0.08;0.05) | 0.73 |
| posterior thalamic radiation | -0.06(-0.10;-0.01) | 0.010 | 0.01(-0.03;0.05) | 0.66 | 0.06(0.02;0.10) | 0.004 | 0.01(-0.03;0.05) | 0.52 |
| **Association tracts** |  |  |  |  |  |  |  |  |
| superior longitudinal fasciculus | -0.08(-0.13;-0.04) | <0.001^*^ | -0.02(-0.06;0.01) | 0.13 | 0.09(0.04;0.14) | <0.001^*^ | 0.00(-0.03;0.02) | 0.84 |
| inferior longitudinal fasciculus | -0.09(-0.14;-0.04) | <0.001^*^ | 0.00(-0.03;0.03) | 0.87 | 0.10(0.06;0.14) | <0.001^*^ | -0.03(-0.06;0.01) | 0.12 |
| inferior fronto-occipital fasciculus | -0.09(-0.13;-0.04) | <0.001^*^ | 0.02(-0.01;0.04) | 0.24 | 0.10(0.06;0.14) | <0.001^*^ | -0.03(-0.06;-0.01) | 0.014 |
| uncinate fasciculus | -0.07(-0.11;-0.02) | 0.002^*^ | 0.01(-0.02;0.04) | 0.67 | 0.05(0.00;0.09) | 0.041 | -0.01(-0.04;0.02) | 0.53 |
| **Limbic system tracts** |  |  |  |  |  |  |  |  |
| cingulate gyrus part of cingulum | -0.01(-0.05;0.04) | 0.71 | -0.01(-0.04;0.03) | 0.74 | 0.03(-0.02;0.08) | 0.28 | -0.03(-0.08;0.01) | 0.15 |
| parahippocampal part of cingulum | -0.03(-0.08;0.02) | 0.22 | -0.02(-0.07;0.02) | 0.23 | 0.03(-0.02;0.08) | 0.27 | -0.02(-0.07;0.03) | 0.50 |
| fornix | -0.07(-0.12;-0.02) | 0.006 | 0.01(-0.04;0.07) | 0.69 | 0.03(0.00;0.07) | 0.050 | 0.00(-0.04;0.05) | 0.82 |
| **Callosal tracts** |  |  |  |  |  |  |  |  |
| forceps major | -0.05(-0.09;-0.01) | 0.013 | 0.00(-0.03;0.04) | 0.94 | 0.05(0.01;0.09) | 0.013 | 0.01(-0.02;0.05) | 0.51 |
| forceps minor | -0.05(-0.09;-0.01) | 0.010 | 0.00(-0.04;0.04) | 0.94 | 0.05(0.00;0.09) | 0.044 | -0.03(-0.06;0.00) | 0.07 |
| The table shows the association of plasma neurofilament light chain (NfL) at baseline with tract-specific white matter integrity at baseline (mean difference), and the association of baseline NfL with change in tract-specific white matter integrity (slope difference). The mean differences represent the difference in standardized white matter integrity markers per standard deviation increase in log transformed plasma NfL level at baseline. The slope differences represent the additional change in standardized white matter integrity markers expressed per 5 years of follow-up. Models are adjusted for age, age^2^, sex, time between blood sampling and neuroimaging, total intracranial volume, tract-specific white matter volume and log-transformed white matter lesion volume of the investigated tract, assay batch number, creatinine, cholesterol, smoking, diabetes mellitus, body mass index and hypertension. CI = confidence interval.  ^*^ =significant at p<0.003 | | | | | | | | |

**Supplementary Table 6**

|  | **Men** | **Women** |  |
| --- | --- | --- | --- |
|  | Mean differences (95%CI) | Mean differences (95%CI) | p-value for interaction |
| Total brain volume | -0.01(-0.05; 0.03) | -0.03(-0.07; 0.01) | 0.55 |
| Gray matter volume | -0.04(-0.10; 0.02) | 0.01(-0.06; 0.07) | 0.10 |
| Hippocampal volume | -0.01(-0.10; 0.08) | -0.02(-0.11; 0.06) | 0.61 |
| Normal appearing white matter volume | -0.01(-0.09; 0.06) | -0.08(-0.14; -0.02) | 0.053 |
| White matter hyperintensities volume | 0.12(0.03; 0.22) | 0.06(-0.04; 0.16) | 0.98 |
| Fractional anisotropy | 0.01(-0.08;0.10) | -0.18(-0.29;-0.06) | 0.25 |
| Mean diffusivity | 0.06(-0.02;0.14) | 0.22(0.12;0.32) | 0.15 |
| Mean cortical thickness | 0.04(-0.06; 0.14) | -0.03(-0.13; 0.08) | 0.73 |
| Mean cortical thickness Mayo AD signature ROI | 0.02(-0.08; 0.13) | -0.07(-0.17; 0.04) | 0.88 |
| Mean cortical thickness Harvard AD signature ROI | 0.06(-0.05; 0.16) | -0.03(-0.13; 0.07) | 0.32 |
|  |  |  |  |
|  | Odds ratio (95%CI) | Odds ratio (95%CI) | p-value for interaction |
| Lacunar infarcts presence | 1.74(1.16;2.60) | 1.30(0.73;2.30) | 0.47 |
| Microbleeds presence | 1.17(0.86;1.58) | 0.77(0.56;1.05) | 0.011 |
| Microbleed factor (1 vs 0) | 1.10(0.74; 1.64) | 0.69(0.47; 1.01) | 0.039 |
| Microbleed factor(>2 vs 0) | 1.20(0.80; 1.82) | 0.91(0.57; 1.46) | 0.17 |
|  |  |  |  |
|  | Slope differences (95% CI) | Slope differences (95% CI) | p-value for interaction |
| Total brain volume | -0.01(-0.02; 0.01) | 0.00(-0.01; 0.01) | 0.66 |
| Gray matter volume | -0.02(-0.05; 0.02) | -0.03(-0.06; 0.01) | 0.45 |
| Hippocampal volume | 0.01(-0.03; 0.04) | 0.03(0.00; 0.07) | 0.39 |
| Normal appearing white matter volume | 0.01(-0.02; 0.04) | 0.02(0.00; 0.05) | 0.48 |
| White matter hyperintensities volume | 0.00(-0.03; 0.03) | 0.00(-0.03; 0.03) | 0.59 |
| Fractional anisotropy | 0.01(-0.03; 0.05) | -0.01(-0.06; 0.04) | 0.76 |
| Mean diffusivity | -0.01(-0.05; 0.03) | 0.01(-0.05; 0.06) | 0.31 |
| Mean cortical thickness | -0.02(-0.06; 0.02) | 0.07(0.03; 0.12) | 0.002^*^ |
| Mean cortical thickness Mayo AD signature ROI | -0.01(-0.06; 0.04) | 0.10(0.04; 0.16) | 0.014 |
| Mean cortical thickness Harvard AD signature ROI | -0.02(-0.07; 0.02) | 0.07(0.02; 0.12) | 0.002^*^ |
| The table shows the association of plasma neurofilament light chain (NfL) at baseline with neuroimaging markers at baseline (mean difference/odds ratio), and the association of baseline NfL with change in neuroimaging markers (slope difference) in men and women separately. The mean differences represent the difference in standardized neuroimaging per standard deviation increase in log transformed plasma NfL level at baseline. The slope differences represent the additional change in standardized neuroimaging markers expressed per 5 years of follow-up. Longitudinal results reflect the change in standardized brain volumes expressed per year of follow-up. Models are adjusted for baseline age, age^2^, sex, time between blood sampling and neuroimaging, intracranial volume, normal appearing white matter (for diffusion tensor imaging analyses only), assay batch number, creatinine, cholesterol, smoking, diabetes mellitus, body mass index and hypertension. CI = confidence interval; NA=not applicable. ^*^ =significant at p<0.006 | | | |

**Supplementary Table 7**

|  | **Below 70 years** | **Above 70 years** |  |
| --- | --- | --- | --- |
|  | Mean differences (95%CI) | Mean differences (95%CI) | p-value for interaction |
| Total brain volume | -0.01(-0.05; 0.02) | -0.04(-0.09; 0.01) | 0.32 |
| Gray matter volume | -0.03(-0.08; 0.02) | 0.01(-0.07; 0.09) | 0.90 |
| Hippocampal volume | -0.02(-0.09; 0.06) | -0.05(-0.16; 0.07) | 0.52 |
| Normal appearing white matter volume | -0.02(-0.08; 0.04) | -0.11(-0.19; -0.02) | 0.29 |
| White matter hyperintensities volume | 0.09(0.01; 0.17) | 0.09(-0.05; 0.23) | 0.97 |
| Fractional anisotropy | -0.10(-0.21;0.00) | -0.07(-0.17;0.03) | 0.52 |
| Mean diffusivity | 0.14(0.05;0.23) | 0.14(0.04;0.24) | 0.72 |
| Mean cortical thickness | 0.00(-0.09; 0.09) | 0.01(-0.13; 0.14) | 0.75 |
| Mean cortical thickness Mayo AD signature ROI | -0.03(-0.12; 0.06) | -0.01(-0.16; 0.13) | 0.96 |
| Mean cortical thickness Harvard AD signature ROI | 0.01(-0.08; 0.10) | 0.01(-0.13; 0.15) | 0.89 |
|  |  |  |  |
|  | Odds ratio (95%CI) | Odds ratio (95%CI) | p-value for interaction |
| Lacunar infarcts presence | 1.45(0.91;2.31) | 1.47(0.94;2.29) | 0.83 |
| Microbleeds presence | 1.03(0.78;1.36) | 0.92(0.66;1.28) | 0.81 |
| Microbleed factor (1 vs 0) | 0.95(0.67; 1.33) | 0.76(0.47; 1.21) | 0.33 |
| Microbleed factor(>2 vs 0) | 1.03(0.66; 1.60) | 1.12(0.73; 1.71) | 0.49 |
|  |  |  |  |
|  | Slope differences (95% CI) | Slope differences (95% CI) | p-value for interaction |
| Total brain volume | -0.01(-0.02; 0.00) | 0.01(-0.02; 0.04) | 0.006 |
| Gray matter volume | -0.03(-0.05; 0.00) | 0.00(-0.07; 0.08) | 0.71 |
| Hippocampal volume | 0.02(-0.01; 0.04) | 0.05(-0.01; 0.11) | 0.27 |
| Normal appearing white matter volume | 0.01(-0.01; 0.04) | 0.01(-0.05; 0.07) | 0.14 |
| White matter hyperintensities volume | 0.01(-0.02; 0.03) | -0.03(-0.07; 0.02) | 0.052 |
| Fractional anisotropy | 0.00(-0.04; 0.03) | 0.03(-0.06; 0.11) | 0.76 |
| Mean diffusivity | 0.00(-0.03; 0.03) | 0.00(-0.10; 0.11) | 0.91 |
| Mean cortical thickness | 0.01(-0.02; 0.05) | 0.08(-0.02; 0.18) | 0.28 |
| Mean cortical thickness Mayo AD signature ROI | 0.04(0.00; 0.08) | 0.05(-0.06; 0.17) | 0.90 |
| Mean cortical thickness Harvard AD signature ROI | 0.01(-0.02; 0.05) | 0.05(-0.06; 0.15) | 0.85 |
| The table shows the association of plasma neurofilament light chain (NfL) at baseline with neuroimaging markers at baseline (mean difference/odds ratio), and the association of baseline NfL with change in neuroimaging markers (slope difference) stratified by baseline age. The mean differences represent the difference in standardized neuroimaging per standard deviation increase in log transformed plasma NfL level at baseline. The slope differences represent the additional change in standardized neuroimaging markers expressed per 5 years of follow-up. Longitudinal results reflect the change in standardized brain volumes expressed per year of follow-up. Models are adjusted for baseline age, age^2^, sex, time between blood sampling and neuroimaging, intracranial volume, normal appearing white matter (for diffusion tensor imaging analyses only), assay batch number, creatinine, cholesterol, smoking, diabetes mellitus, body mass index and hypertension. CI = confidence interval, ROI = region of interest. ^*^ =significant at p<0.006 | | | |

**Supplementary Table 8**

|  | **Cross-sectional Old DTI** |  | **Cross-sectional New DTI** |  |
| --- | --- | --- | --- | --- |
| **Model I** | Mean differences (95%CI) | p-value | Mean differences (95%CI) | p-value |
| Fractional anisotropy | -0.10(-0.16;-0.04) | 0.002 | -0.10(-0.15;-0.06) | <0.001 |
| Mean diffusivity | 0.11(0.06;0.17) | <0.001 | 0.08(0.04;0.12) | <0.001 |
| **Model II** | Mean differences (95%CI) | p-value | Mean differences (95%CI) | p-value |
| Fractional anisotropy | -0.07(-0.14;0.00) | 0.049 | -0.10(-0.15;-0.05) | <0.001 |
| Mean diffusivity | 0.12(0.06;0.19) | <0.001 | 0.07(0.03;0.12) | 0.001 |
| The table shows the association between plasma neurofilament light chain (NfL) at baseline and diffusion tensor imaging (DTI) measurements assessed with an old and new protocol. Model I is adjusted for baseline age, a non-linear term of baseline age, sex, time between blood sampling and DTI imaging, batch number, normal appearing white matter volume and total intracranial volume. Model II is additionally adjusted for creatinine, cholesterol, smoking, diabetes mellitus, body mass index and hypertension. DTI = diffusion tensor imaging, CI = confidence interval | | | | |

**Supplementary Table 9. Association between plasma neurofilament light chain level and neuroimaging markers in persons without a history of clinical stroke.**

|  | **Cross-sectional** |  | **Longitudinal** |  |
| --- | --- | --- | --- | --- |
|  | Mean differences (95%CI) | p-value | Slope differences (95%CI) | p-value |
| Total brain volume | -0.02(-0.05;0.01) | 0.27 | 0.00(-0.01;0.01) | 0.42 |
| Gray matter volume | -0.02(-0.07;0.02) | 0.34 | -0.02(-0.05;0.01) | 0.12 |
| Hippocampal volume | -0.01(-0.08;0.05) | 0.66 | 0.03(0.00;0.05) | 0.035 |
| Mean cortical thickness | 0.00(-0.07;0.08) | 0.92 | 0.03(0.00;0.06) | 0.09 |
| Mean cortical thickness Mayo AD signature ROI | -0.02(-0.10;0.05) | 0.54 | 0.04(0.00;0.08) | 0.028 |
| Mean cortical thickness Harvard AD signature ROI | 0.01(-0.06;0.08) | 0.77 | 0.02(-0.01;0.06) | 0.15 |
| Normal appearing white matter volume | -0.03(-0.08;0.02) | 0.20 | 0.02(-0.01;0.04) | 0.18 |
| White matter hyperintensities volume | 0.07(0.00;0.14) | 0.047 | 0.00(-0.02;0.02) | 0.99 |
| Fractional anisotropy | -0.08(-0.15; -0.01) | 0.033 | 0.00(-0.03;0.03) | 0.97 |
| Mean diffusivity | 0.13(0.07; 0.20) | <0.001^*^ | 0.00(-0.04;0.03) | 0.83 |
|  |  |  |  |  |
|  | Odds ratio (95%CI) |  |  |  |
| Lacunar infarcts presence | 1.45(1.02; 2.05) | 0.037 |  |  |
| Microbleeds presence (any vs. none) | 0.91(0.73; 1.14) | 0.42 |  |  |
| Microbleed factor (1 vs. 0) | 0.87(0.66; 1.14) | 0.31 |  |  |
| Microbleed factor(≥2 vs. 0) | 0.97(0.71; 1.33) | 0.84 |  |  |
| The table shows the association of plasma neurofilament light chain (NfL) at baseline with neuroimaging markers at baseline (mean difference/odds ratio), and the association of baseline NfL with change in neuroimaging markers (slope difference), excluding participants with a clinical stroke before their baseline scan or during follow-up. The mean differences represent the difference in standardized neuroimaging per standard deviation increase in log transformed plasma NfL level at baseline. The slope differences represent the additional change in standardized neuroimaging markers expressed per 5 years of follow-up. Longitudinal results reflect the change in standardized brain volumes expressed per year of follow-up. Models are adjusted for age, age^2^, sex, time between blood sampling and neuroimaging, intracranial volume, normal appearing white matter (for DTI analyses only), assay batch number, creatinine, cholesterol, smoking, diabetes mellitus, body mass index and hypertension. CI = confidence interval, ROI = region of interest. ^*^ =significant at p<0.006 | | | | |

**Supplementary References**

1. Wieberdink, R.G., et al., *Trends in stroke incidence rates and stroke risk factors in Rotterdam, the Netherlands from 1990 to 2008.* European Journal of Epidemiology, 2012. **27**(4): p. 287-295.

2. Ikram, M.A., et al., *The Rotterdam Scan Study: design update 2016 and main findings.* Eur J Epidemiol, 2015. **30**(12): p. 1299-315.

3. Vrooman, H.A., et al., *Multi-spectral brain tissue segmentation using automatically trained k-Nearest-Neighbor classification.* Neuroimage, 2007. **37**(1): p. 71-81.

4. de Boer, R., et al., *White matter lesion extension to automatic brain tissue segmentation on MRI.* Neuroimage, 2009. **45**(4): p. 1151-61.

5. Vernooij, M.W., et al., *Incidental findings on brain MRI in the general population.* N Engl J Med, 2007. **357**(18): p. 1821-8.

6. Koppelmans, V., et al., *Global and focal white matter integrity in breast cancer survivors 20 years after adjuvant chemotherapy.* Hum Brain Mapp, 2014. **35**(3): p. 889-99.

7. Smith, S.M., et al., *Tract-based spatial statistics: voxelwise analysis of multi-subject diffusion data.* Neuroimage, 2006. **31**(4): p. 1487-505.

8. Jenkinson, M. and S. Smith, *A global optimisation method for robust affine registration of brain images.* Med Image Anal, 2001. **5**(2): p. 143-56.

9. Vernooij, M.W., et al., *White matter microstructural integrity and cognitive function in a general elderly population.* Arch Gen Psychiatry, 2009. **66**(5): p. 545-53.

10. de Groot, M., et al., *Tract-specific white matter degeneration in aging: the Rotterdam Study.* Alzheimers Dement, 2015. **11**(3): p. 321-30.
